# Factors affecting knowledge of National Health Insurance Policy among out-patients in Lao PDR: an exit interview study

**DOI:** 10.1080/16549716.2020.1791414

**Published:** 2020-08-03

**Authors:** Kongmany Chaleunvong, Bounfeng Phoummalaysith, Bouaphat Phonvixay, Vanpahnom Sychareun, Jo Durham, Dirk R. Essink

**Affiliations:** a Institute of Research and Education Development, University of Health Sciences, Vientiane, Lao PDR; b Lao National Health Insurance, Ministry of Health, Vientiane, Lao PDR; c School of Public Health, Queensland University of Technology, Brisbane, Australia; d Athena Institute for Research on Innovation and Communication in Health and Life Sciences, Faculty of Earth and Life Sciences, Vrije Universiteit, Amsterdam, Netherlands

**Keywords:** LEARN: Sexual Reproductive Health, ANC and Nutrition, National health insurance, universal health coverage, out-of-pocket payment, patient knowledge, exit survey

## Abstract

**Background:**

Universal health coverage is target 3.8 of the Sustainable Development Goals. In many lower-middle-income countries, however, major coverage gaps exist. Those who do receive services often experience high out-of-pocket expenses. To achieve universal health coverage, Lao PDR, a lower-middle-income country in South-East Asia, is shifting from a fragmented model of health financing to a national health insurance scheme.

**Objective:**

The objective of this cross-sectional survey was to assess the knowledge of the NHI in Lao PDR among insured in- and out-patients using health services at selected public health facilities at provincial, district and health centre level in six provinces.

**Methods:**

This was a cross-sectional survey. Healthcare facilities were selected based on the rate of use of health services at the health facility and participants selected using systematic random sampling. Exit interviews were conducted with in- and out-patients of each selected health facility, using a pre-tested structured questionnaire. Descriptive statistics were generated including means (median), frequency and percentages. Poisson regression was applied to determine the factors associated with knowledge of the insurance scheme.

**Results:**

In total 326 participants were recruited (response rate 93%). Of these, less than two-thirds (62.3%) said they had their eligibility documents with them. Only 23.6% knew the co-payment fee at the health centre level; while 18.1% and 18.7% knew about the co-payment fee at the district and provincial healthcare level, respectively. A key determinant of accessing NHI and health services was knowledge of the scheme and its benefits.

**Conclusion:**

This study suggests in Lao PDR, awareness about health insurance is low. More innovative demand-side strategies are needed to create awareness and understanding of the NHI and its benefits. Without an understanding of what insurance policies mean, universal health coverage cannot be achieved, even where appropriate and acceptable services are accessible.

## Background

Universal health coverage (UHC); that is, all people receive the essential health services they need, without suffering financial hardship is target 3.8 of the Sustainable Development Goals (SDG). In many lower-middle-income countries (LMCs), however, major coverage gaps persist. Further, those who access services often experience high out-of-pocket (OOP) expenses. High OPP expenses are a disincentive for people to access the care they need and can be catastrophic for poorer households [1–6]. Achieving UHC requires adequate and sustainable funding. Commonly, governments raise sustainable funding for UHC through prepaid revenues. Prepaid revenues derive from a range of sources, including general and earmarked taxes, and can be kept in separate funds or pooled. Pooling is more effective in achieving UHC because it allows the creation of a large, diverse risk-pool allowing health systems to cross-subsidy from the healthy population to those who are sick [6–8].

Within South-East Asia, governments typically rely on a mix of health financing mechanisms including allocations from national budgets, social health insurance, varying degrees of OOP expenses and international donor support [7,8]. The drive to UHC, however, means many countries are reviewing existing financing modalities and enacting healthcare reforms. Within this context, the Lao PDR, a lower-middle-income country with high OOP expenses for health, is shifting from a fragmented risk-sharing model with separate schemes for civil servants, enterprise sector workers, informal sector including the free policy for MCH and the poor to a national insurance scheme (NHI) [9]. As part of this process, the government is merging existing schemes (compulsory social health insurance scheme for civil servants and formal-sector employees, health equity funds for the poor, free maternal and child health, free delivery and free care for children under 5 years old and voluntary community-based health insurance) bringing them under an NHI fund [10]. In its first phase, the NHI targets the informal sector given they are often exposed to more risks than formal sector workers and are often in poorly paid and insecure work, often with delays in presentation and/or are lost to follow up.

The NHI was first launched in the southern province of Attapeu in August 2016 and subsequently scaled up to further three provinces, with the scheme expanded to a total of 17 provinces by the end of 2017 (excluding Vientiane Capital) [11]. Under the scheme, all citizens contribute a small co-payment at point of service except the poor, pregnant women and children under five, with the government further reimbursing facilities. A key determinant for those enrolled in the scheme in accessing health services is knowledge of the scheme and its benefits [1,2,12]. The purpose of the current study was to assess the knowledge of those enrolled in the NHI scheme in Lao PDR.

## Methods

This was a quantitative, cross-sectional descriptive study examining the knowledge of those enrolled in the Laotian NHI scheme. The sample was out- and in-patient patients using health services at selected health facilities at three levels: provincial hospitals (PH), district hospitals (DH) and health centres (HC) in six provinces. The six provinces were Saravan, Attapeu and Sekong in the southern part of the country; Borikhamxay in the central part of the country; and Xieng Khouang and Luang Namtha in the northern part of Lao PDR.

### Sample size and sampling

Four health centres, two district hospitals in each province were selected based on the rate of use of health services at the health facility. The calculated sample size for out-patients was 312 with an additional 10%, making the total 342 participants. This was calculated to allow for multiple regression α = 0.05, Power = 0.8; U = number of independent variables intended = 5 and R = expected multiple correlation coefficient = 0.2.

Systematic random sampling was used to select in- and out-patients of each selected health facility for an exit interview (four health centres, two district-level hospitals one provincial-level hospital). In selecting the sample, the first in-patient participant was selected at random and subsequently, using a sampling interval of two, other in-patient participants were selected systematically, that is, every second patient/caregiver exiting the consultation room was invited to participate in the study before being discharged from the hospital. Out-patients or caregivers were selected in front of the pharmacy unit, applying the same systematic random sample method and sampling interval of two used for in-patients.

Face-to-face exit interviews were conducted using a structured survey tool developed for this study and in collaboration with experts from the Swiss Red Cross and the NHI. Survey questions included socio-demographic characteristics of patients, type of health insurance scheme such as OOP, co-payment only, co-payment with other, exemption payment, type of NIH users such as in or out-patients, provision of NHI eligibility documents to health facility staff, knowledge of the NHI policy. Knowledge questions related to the NHI policy including the co-payment fee schedule for in- and out-patients at the different service levels (health centre’s district and provincial hospitals), and whether participants had their documents with them or not and if the health facility staff had asked to see them.

For the knowledge of the co-payment, fee schedule for OPD at correct responses was coded as HC = 5000 LAK, DH 10,000 LAK, PH 15,000 LAK and for IPD- HC = 5000 LAK, DH and PH LAK 30. All knowledge questions were summed, with the knowledge score ranging from 0 to 10. The higher the score the higher the level of knowledge. The survey instrument was piloted in Vientiane Province, with revisions made based on feedback. All team members were trained in the administration of the survey prior to administration. The final survey tool was entered into a tablet using CsPro application version 7.1.

### Data analysis

Data were cleaned and analysed using STATA 13.1. Descriptive statistics were generated including means (median), frequency and percentages. Additionally, Poisson regression was applied to determine the factors associated with knowledge of NHI as count data.

## Ethical approval

Ethical approval was received from the National Ethical Committee for Health Research of Lao PDR. Verbal informed consent (approved by the Ethical Committee) was gained from each respondent by the interviewer prior to beginning the interview. All identifiable data were removed from the questionnaire prior to analysis.

## Results

Three hundred and twenty-six participants were recruited into the study with a response rate of 93%. Table 1 shows that 116 (35.6%) of respondents were aged below 18, 175 (53.7%) of respondents were aged 18 to 59 years and 35 (10.7%) were equal to and above 60 years of age. The mean age of respondents was 45.2 (1.6) years. In total, 141 respondents (43.4%) were males; 185 (56.8%) were females. All respondents worked in the informal sector. Two-fifths of respondents (40.5%) lived 1–5 km from the health facility.

**Table 1.** Characteristic of the in-patients and out-patients respondents.

	Out-patients					
	PH		DH/HC		TOTAL	
NHI Users	N 111	%	N 215	%	N 326	**%**
**Province**						
Xieng Khouang	20	18.0	14	6.5	34	10.4
Luang Namtha	20	18.0	34	15.8	54	16.6
Borikhamxay	42	37.8	81	37.7	123	37.7
Saravan	11	9.9	25	11.6	36	11.0
Sekong	5	4.5	26	12.1	31	9.5
Attapeu	13	11.7	35	16.3	48	14.7
**Age group**						
<1 yr	1	0.9	12	5.6	13	4.0
1–5 yrs	17	15.3	42	19.5	59	18.1
6/17 yrs	15	13.5	29	13.5	44	13.5
18/59 yrs	59	53.2	116	54.0	175	53.7
≥ 60 yrs	19	17.1	16	7.4	35	10.7
Median		31		24		
Min		<1		<1		
Max		90		88		
**Sex**						
Male	69	62.2	116	54.0	185	56.7
Female	42	37.8	99	46.0	141	43.3
**Occupation of patients**						
Self employed	26	23.4	18	8.4	44	13.5
Work for Family (no salary)	13	11.7	26	12.1	39	12.0
Unemployed	37	33.4	116	53.9	153	46.9
Student	5	4.5	6	2.8	11	3.4
Other, specify	30	27.0	49	22.8	79	24.2
Do you/patient live in this province and district?						
Yes	95	85.6	210	97.7	305	93.6
No	16	14.4	5	2.3	21	6.4
How far from you living place to this health facility?						
Less than 1 km	2	1.8	42	19.5	44	13.5
1–5 kms	36	32.4	96	44.7	132	40.5
6–10 kms	11	9.9	27	12.6	38	11.7
11–30 kms	29	26.1	20	9.3	49	15.0
More than 30 kms	19	17.1	8	3.7	27	8.3
UK	14	12.6	22	10.2	36	11.0
Median		7		3		
Min		<1		<1		
Max		200		95		

### Patients with NHI eligibility documents

Table 2 shows whether patients were able to provide their NHI eligibility documents. As seen in Table 2, more than four-fifths of participants (83.8%) had their eligibility documents with them and were able to show them when asked; slightly more than one-fifth (21.5%), showed their eligibility documents without being asked.

**Table 2.** NHI eligibility documents.

	Out-patients					
Eligibility documents	PH		DH/HC		Total	
Asked to provide NHI eligibility documents	N 111	%	N 215	%	N 326	%
Showed documents when asked by facility staff	75	67.6	128	59.5	203	62.3
Showed documents without being asked by facility staff	18	16.2	52	24.2	70	21.5
Asked by facility for documents but did not bring the documents	10	9.0	16	7.4	26	8.0
Asked by facility staff but did not have the document	3	2.7	13	6.0	16	4.9
Were not asked by facility staff and did not bring the document	3	2.7	3	1.4	6	1.8
Were not asked by facility staff and did not have the document	2	1.8	3	1.4	5	1.5

### Patients’ knowledge of NHI policy

As seen in Table 3, overall, participants had low levels of knowledge regarding the scheme and fee schedule. Only 23.9% of out-patients, for example, knew the co-payment fee at the health centre level, while 18.1% and 18.7% knew about the co-payment fee at the district and provincial healthcare level, respectively. Of the included in-patients, 15.6% knew the level of co-payment at the health centre level, while 11.3% and 13.5% knew the co-payment fee relevant to the level of care. Four in five participants (81.0%) understood the NHI eligibility documents.

**Table 3.** Knowledge on NHI policy of respondent’s out-patient.

NHI Users	Out-patients					
	PH		DH/HC		TOTAL	
NHI Users	N 111	%	N 215	%	N 326	%
Know about fee schedule of co-payment for OPD in HC						
Wrong answer	92	82.9	156	72.6	248	76.1
Correct answer	19	17.1	59	27.4	78	23.9
Know about fee schedule of co-payment for OPD in DH						
Wrong answer	96	86.5	171	79.5	267	81.9
Correct answer	15	13.5	44	20.5	59	18.1
Know about fee schedule of co-payment for OPD in PH						
Wrong answer	62	55.9	203	94.4	265	81.3
Correct answer	49	44.1	12	5.6	61	18.7
Know about fee schedule of co-payment for IPD in HC						
Wrong answer	97	87.4	178	82.8	275	84.4
Correct answer	14	12.6	37	17.2	51	15.6
Know about fee schedule of co-payment for IPD in DH						
Wrong answer	96	86.5	193	89.8	289	88.7
Correct answer	15	13.5	22	10.2	37	11.3
Know about fee schedule of co-payment for IPD in PH						
Wrong answer	79	71.2	203	94.4	282	86.5
Correct answer	32	28.8	12	5.6	44	13.5
Understanding of NHI eligibility document (Multiple answers)						
ID card	6	6.9	18	9.9	24	7.4
Family book	86	69.5	178	78.4	264	81.0
Certificate from village chief	9	6.6	15	9.5	24	7.4
Health book/card	13	34.3	29	24.4	42	12.9

**Figure 1.**
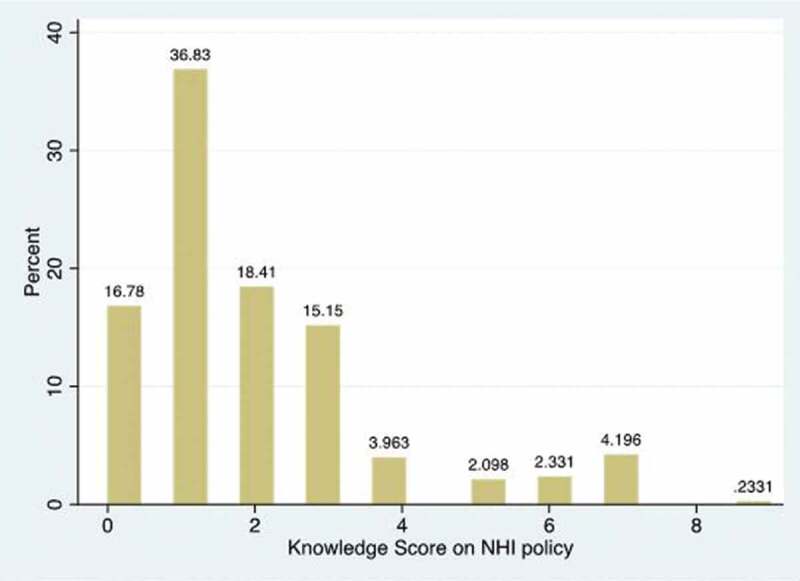
Knowledge score on NHI policy among out and in-patients.

Figure 1 shows the NHI policy knowledge score among out-patients based on the 10 knowledge questions being summed up with the score ranging from 0 to 10, with 10 being the highest. Many patients (36.8%) had knowledge score of 1 on NHI policy, while 4.2% had a knowledge score 7.

### Factors related to knowledge of NIH policy

Table 4 presents factors related to knowledge of the NHI scheme among out-patients (Poisson regression analysis). Factors significantly associated with knowledge of the scheme were type of health insurance (RR: 1.5; 95%CI: 1.5–3.5); purchasing medications or supplies outside the health facility (RR: 1.9; 95% CI: 1.3–2.8); asking to provide NHI eligibility documents by facility staff (RR: 1.3; 95% CI: 1.1–1.6); showing the documents (RR: 1.5; 95% CI: 1.2–2.0).

**Table 4.** Factors related to knowledge of NIH policy among out-patients (Poisson regression analysis).

	Knowledge on NHI policy						
	Statistics value			Crude		Adjusted	
Factor	Median	Min	Max	IRR	95%CI	IRR	95% CI
Facility use							
DH/HC	1	0	7	1		1	
PH	2	0	7	1.2	1.0–1.4	1.3	1.1–1.5
Type of payment							
OOP	1	0	6	1		1	
Exemption payment	1	0	7	1.2	0.9–1.6	1.5	1.1–2.0
Co-payment only	2	0	7	`1.3	1.0–1.6	1.7	1.3–2.3
Co-payment with other cost	2	0	7	1.7	1.2–2.5	2.3	1.5–3.5
Are you a in-patient?							
No	1	0	7	1			
Yes	2	0	7	1.2	1.1–1.4		
Age of patients							
≤ 5 yr	1	0	7	1			
6–17 yrs	1	0	7	0.8	0.6–1.1		
≥ 18 yrs	1.5	0	7	1.0	0.8–1.2		
Gender of patient							
Female	1	0	7	1			
Male	1	0	7	0.9	0.8–1.1		
Occupation of patients							
Unemployed	1	0	7	1		1	
Self-employed/student	2	0	7	1.3	1.1–1.5	1.3	1.1–1.5
Do you/patient live in this province and district?							
Yes	1	0	7	1			
No	1	0	7	1.0	0.7–1.4		
How far from you living place to this health facility?							
≤ 5 kms	1	0	7	1			
>5 kms	1.5	0	7	1.1	0.9–1.3		
Purchasing some medicines or supplies outside this health facility							
No	1	0	7	1		1	
Yes	2	0	7	1.6	1.1–2.3	1.8	1.2–2.6
Asking to provide NHI eligibility documents by facility staffs?							
Yes	1	0	7	1		1	
No	1	0	7	1.2	1.0–1.4	1.2	1.0–1.5
Showing the documents							
Yes	1	0	7	1		1	
No, I do not bring the documents	2	0	7	1.1	0.9–1.4	1.5	1.2–2.0
No, I do not have the documents	1	0	3	0.6	0.3–1.0	0.7	0.4–1.2

No association was found related to socio-demographic variables or level of healthcare and knowledge.

## Discussion

Overall, understanding of the benefits of the NHI, the limits of coverage and co-payment policies were low. Most participants had brought their eligibility documents with them and were able to present them to health facility staff. However, a few participants forgot to bring their eligibility documents or may have lost or misplaced them. Suboptimal understanding of the NHI and/or not having the relevant documents may prevent people from obtaining the health services they need and may result in patients being charged additional informal fees [2].

A lack of awareness may be due to limited community consultation and inadequate information dissemination processes in relation to the NHI. Studies in other developing countries have also shown a low level of awareness of health insurance especially among the informal sector of the population [1,13]. This study also suggests the need for more interventions to increase knowledge among NHI cardholders. A better understanding of the NHI benefits will allow enrolees to make more make informed decisions about when, and whether, to access healthcare services. Studies elsewhere show when people understand their health insurance and co-payments, they are more likely to use appropriate health services when needed [14–16].

While overall level of knowledge was generally low, some differences in levels of knowledge were observed. Patients who came with and presented the relevant documents at the health facility and patients who were eligible to be exempt from payment demonstrated 1.5 times and 1.3 times more knowledge than patients with OOP expenses. For those who were exempt from co-payment their knowledge may be based on knowledge of the earlier health equity fund under which patients below a certain level of income were exempt from payment or health equity funds for the poor or understood the free maternal and child health policy, given most of the participants were women. It is not clear from this study, however, if participants included in the study understood that the insurance scheme had changed.

Patients with co-payment and other costs had 2.5 times more knowledge than patients with OOP. This may be because patients with OOP and no subsidy from the NHI were those with no insurance. Patients who bought some medicine or supplies outside of the health facility that were not available within the hospitals had 1.9 times more knowledge than patients who did not buy medicine outside of the health facility. This because these patients understood the benefit packages under the NHI and were able to make more informed decisions about purchasing additional medications not available in the hospitals.

Our findings indicate the need for more awareness-raising interventions among enrolled families to maximize efforts to achieve UHC. To improve understanding of the NHI scheme, a comprehensive and intensive health education and promotion program using multiple strategies to reach informal workers should be implemented. Strategies may include dissemination of information through credible communicators such as village chiefs in community meetings and local healthcare staff. This is a commonly used approach in Lao PDR, especially in rural settings. Other strategies could include using local media such as village speaker, radio, print and television which have been found to be effective elsewhere [17]. Village health workers and volunteers could also serve as additional sources of information about the scheme, its benefits and how it works. Without increasing awareness of the conditions and benefits of the NHI, patients may continue to pay more than they should, and may be exposed to financial hardship or not purchase other recommended medicines that are outside of the NHI. Concern about high OPP may also prevent people from using appropriate preventive, curative, rehabilitative and palliative healthcare services in a timely manner [2].

Studies in similar settings have also recommended demand-side interventions to increase awareness of NHI policies, coverage and benefits to improve utilization [18,19]. Information boards about the NHI and scheduled co-payments could be displayed in all healthcare facilities included in the scheme. In addition, programs working in health that disseminate information to informal workers could include messages related to the NHI in their programming, with messages and materials designed to reach both literate and non-literate participants [18]. Increasing awareness of the scheme will reduce the predominant OOP method and move Lao PDR further towards a prepayment method for healthcare. In turn, this will minimise potentially harmful coping strategies such as borrowing to pay for healthcare expenses or late presentation, both of which can be catastrophic for some families.

## Limitations

A limitation of this study is it cannot be generalised beyond included study sites. Further, we only included people who had sought care and did not interview people who may have needed healthcare but did not seek it, or those who are enrolled in the NHI but used private services not included in the NHI scheme. Nevertheless, the response rates were high, and the study provides some important pointers for Lao PDR in expanding the NHI. Also, important is the interviews were conducted at the hospital premises and this might have introduced some bias. To minimize bias we ensured participants’ informed consent and awareness that confidentiality would be protected. Finally, while the findings are specific to the Lao PDR, they are likely to be relevant to other countries in low-income settings with a large informal workforce that are instituting health financing reform and NHI schemes to achieve universal health coverage.

## Conclusion

This study reveals in Lao PDR awareness about health insurance is low. The study findings suggest more innovative demand-strategies are needed to create awareness and understanding of the NHI and its benefits. Without an understanding of what insurance policies mean, universal health coverage cannot be achieved even where appropriate and acceptable services are accessible. Thus, universal coverage and access to appropriate and accessible health services necessarily go hand-in-hand.

## Paper context

Achieving universal health coverage is target 3.8 of the Sustainable Development Goals. To achieve this target, Lao PDR is implementing a new national health insurance scheme, initially targeting the informal workforce. Our study suggests awareness about health insurance scheme is low and enrolees have limited knowledge of eligibility criteria, benefits and co-payment, potentially limiting demand. Innovative strategies to create awareness and understanding are needed to increase the informal workforce’s timely use of needed health services.
